# Differential regulation and preventive mechanisms of green tea powder with different quality attributes on high-fat diet-induced obesity in mice

**DOI:** 10.3389/fnut.2022.992815

**Published:** 2022-09-29

**Authors:** Jin Wang, Lu Dong, Jia-qiang Hu, Yuan-yi-fei Wang, Ang Li, Bo Peng, Bo-wei Zhang, Jing-min Liu, Shuo Wang

**Affiliations:** Tianjin Key Laboratory of Food Science and Health, School of Medicine, Nankai University, Tianjin, China

**Keywords:** different qualities of green tea powder, prevent obesity, gut microbiota, modulatory effects, mechanisms

## Abstract

Tea powder has been reported to have some physiological functions. However, there is no report on whether there are differences in the active ingredients of tea powder with different qualities and whether there are different prebiotic mechanisms. This study was aimed to investigate the effects of different qualities of tea powder on preventing obesity from different aspects, namely antioxidation, inflammation, lipid-lowering, and intestinal flora, using an obesity mouse model. The results showed that all three types of tea powder with different qualities could reduce body weight and decrease serum TC, TG, and LDL-C. However, tea powder with different quality attributes exhibited diverse modulatory effects and mechanisms. Tender tea powder contained more tea polyphenols, and it had a better effect on improving oxidative stress. Tender tea powder significantly decreased the abundances of *Blautia*, *Bilophila*, and *Oscillibacter*, and increased the abundances of *Alloprevotella*, *Lachnoclostridium*, *Romboutsia*, and *Ruminococcaceae*_UCG-004. Coarse tea powder contained more dietary fiber, and had a better effect on reducing the food intake and improving lipid metabolism, which could reduce lipid synthesis and increase lipid β-oxidation. Coarse tea powder significantly decreased the abundance of *Dubosiella* and increased the abundances of the *Lachnospiraceae*_NK4A136 group and *Coriobacteriaceae*_UCG-002. Our findings provide a theoretical reference for the comprehensive utilization of tea powder.

## Introduction

Green tea is a type of unfermented tea and has health benefits (1). Many population interventions and epidemiological surveys have reported that drinking tea may increase energy expenditure, reduce body weight, and promote fat oxidation; thus preventing the occurrence and development of obesity (2). Meanwhile, the composition and function of gut microbes have been implicated closely in diseases such as obesity, related metabolic disorders, and inflammation (3). Intestinal flora regulation through dietary factors is an effective strategy that can be used to influence host health.

The quality of tea powder is mostly assessed by its tenderness. Tea powders with different degrees of tenderness have different qualities. Generally, people tend to choose tender and fresh tea, believing that tender tea has higher quality, while coarse tea has poor sensory quality and taste. Coarse tea leaves and their by-products from the processing industry are generally discarded as worthless crop products, and not fully utilized. However, the health benefits of coarse tea cannot be ignored. Some studies have shown that coarse tea contains more tea polysaccharides and relieves diabetes and other metabolic diseases (4, 5). In the meantime, dietary fiber rich in coarse tea can also improve the dynamic balance of the intestinal flora. However, there is no report on whether there is a discrepancy in the active substance composition and content of different qualities of tea powder, and even whether different qualities of tea powder have diverse modulatory effects and mechanisms.

The main active constituents of green tea powder include tea polyphenols, dietary fiber, and amino acids. Tea powder has some physiological functions, such as antimicrobial activity against many microorganisms (6). Our previous study indicated that green tea powder could modulate gut microbiota and improve lipid metabolism in a hyperlipidemia animal model (7). Green tea polyphenols have antioxidant and radical scavenging activity (8). Green tea polyphenols include principally four types of catechins, namely epigallocatechin gallate (EGCG), epicatechin (EC), epicatechin gallate (ECG), and epigallocatechin (EGC) (9). Green tea dietary fiber was demonstrated to reduce blood sugar levels and regulate immunity (10). Green tea dietary fiber cannot be digested and absorbed by small intestine, and thus directly reach the large intestine where it could be fermented by intestinal microbiota (11). Green tea dietary fiber can act as a prebiotic to exert health benefits, which can regulate the gut microbiota, including promoting the growth of the beneficial bacteria and inhibiting the proliferation of the harmful bacteria. Green tea amino acids directly affect the quality and briskness of taste (12).

This purpose of the study was to explore the effects of different qualities of tea powder on preventing and alleviating obesity from different aspects, namely, antioxidation, reduction of inflammation, lipid-lowering effects, and regulation of the intestinal flora, using an obese mouse model. We also tried to clarify the preventive mechanisms of different qualities of green tea powder on obesity. The results of this research could provide a scientific reference for the comprehension of tea and the application of green tea powder with different qualities as a functional food.

## Materials and methods

### Determination of the principal components of green tea powder

Three types of green tea from different harvest periods were produced in Hangzhou. Green tea powder was ground and sieved with a 40-mesh sieve. Tea powder with different qualities were rated and collected. The tender tea group has a high proportion of buds and leaves, such as one or two leaves and a bud. Coarse tea was harvested late, relatively mature, and characterized by fewer tea leaves and more stalks. Medium-tender tea leaves are those whose tenderness is between the coarse and tender tea. The total dietary fiber, insoluble dietary fiber, soluble dietary fiber, cellulose, hemicellulose, lignin, pectin, and total phenol contents were measured with reference to previous methods (7). High-performance liquid chromatography was used to determine the theanine and catechins (EGC, EC, EGCG, ECG) contents. The free amino acids of tea were determined according to GB/T 8314-2013.

### Animals

C57BL/6J mice (8 weeks) were purchased and housed in the Animal Experimental Center of Nankai University. All mice were house in a controlled environment with room temperature 25°C and relative humidity of 50% in 12 h light-dark cycle. The condition of free access to food and water was given in the study. After a week of acclimatization, 35 male mice were randomly divided into five groups (*n* = 7). A standard chow low-fat diet (LFD) was used to feed in the blank control (CK) group, which contained 10% calories from fat (D12450). A high-fat diet (HFD) was used to feed in the model control (F) group, which contained 60% calories from fat (D12492). Another three groups were also fed an HFD but were also intragastrically administered 150 mg/kg/day of tender tea powder (T), medium tender tea powder (M), and coarse tea powder (C) for 8 weeks by intragastric gavage. Green tea powder was administered to mice in the form of a suspension. An equal volume of saline was given in CK and F groups. For animals in the oral gavage groups, the study was administered once daily by gavage. The dose of 150 mg/kg/day was based on the recommended daily dose of dry tea for adults, which was 9 g/day for a 60 kg adult (13). The whole experiment lasted 8 weeks. The body weight and feed intake were measured and recorded every 7 days. After 8 weeks of intervention, the mice were sacrificed painlessly after 12 h fasting. The blood, liver tissue, colon tissue, and cecal content of the mice were carefully collected. The fat tissues, including epididymal fat and perirenal fat, were isolated and weighted. All samples were stored at –80°C for future analysis. The cecal content was used to conduct the bacterial community analysis.

### Histopathology analysis

The liver and colon tissue samples were fixed in 10% formalin solution, and the samples were embedded in paraffin. The histological sections were sliced into 5–10 μm sizes. Hematoxylin and eosin was used for staining the tissue sections to observation using an optical microscope.

### Serum biochemical analysis

The contents of total cholesterol (TC), triglyceride (TG), low-density lipoprotein cholesterol (LDL-C), high-density lipoprotein cholesterol (HDL-C) in serum were measured with commercial kits (Nanjing Jiancheng Bioengineering Institute). The content of tumor necrosis factor-alpha (TNF-α), lipopolysaccharides (LPS), and interleukin-6 (IL-6) was determined by commercial ELISA kits (Nanjing Jiancheng Bioengineering Institute).

### Oxidative stress associated indicator analysis in the liver and colon of high-fat diet induce obesity mice

Glutathione (GSH), malondialdehyde (MDA), Superoxide dismutase (SOD), and catalase (CAT) were used to assess the liver and colon oxidative stress levels of the mice using commercial kits (Nanjing Jiancheng Bioengineering Institute, Nanjing, China).

### Real-time quantitative PCR for the expression of inflammatory and lipid metabolism-related genes

The RNA extraction and the cDNA synthesis in the liver and colon tissue referred to previous methods (7). Total RNA was extracted from the tissues (50–100 mg) following the kit’s protocol, measured by nanodrop, and finally reversed to cDNA. Real-time PCR was measured using the SYBR green master mix (Vazyme). The PCR conditions were as follows: (1) Heating 95°C for 30 s; (2) 40–45 cycles at 95°C for 10 s and 60°C for 30 s; (3) 60–90°C for 5 s. The specific primer sequences are shown in Table 1. β-actin gene was as the normalization. Cq was used to calculate the relative expression of the target gene by the 2**^–^**^Δ^ ^Δ^ *^Cq^* method. Cq value referred to the number of amplification cycles corresponding to when the fluorescence signal of the amplification product reached the set fluorescence threshold during the qPCR amplification process.

**TABLE 1:** Specific primer sequences used for the real-time quantitative PCR.

Gene	Forward primer	Reverse primer
IL-6	CTGCAAGAGACTTCCATCCAG (21 bp)	AGTGGTATAGACAGGTCTGTTGG (23 bp)
IL-1β	GAAGAAGAGCCCATCCTCTG (20 bp)	GTTCATCTCGGAGCCTGTAG (20 bp)
TNF-α	CAGGCGGTGCCTATGTCTC (19 bp)	CGATCACCCCGAAGTTCAGTAG (22 bp)
PPARα	TGCAGCCTCAGCCAAGTTGAA (21 bp)	TCCCGAACTTGACCAGCCA (19 bp)
FAS	GAGGGTGTGCCATTCTGTCA (20 bp)	GCTATTCTCTACCGCTGGGG (20 bp)
PPAR*γ*	CTGGGGTATTGGGTCGC (17 bp)	GCTTCTTTCAAATCTTGTCTGTCAC (25 bp)
SREBP1c	CTGGTGAGTGGAGGGACCAT (20 bp)	GACCGGTAGCGCTTCTCAAT (20 bp)
CPT1	TGGCATCATCACTGGTGTGTT (21 bp)	GTCTAGGGTCCGATTGATCTTTG (23 bp)
LXR	AATGAAGCTGGTGAGCCTCC (20 bp)	CCATGTGGCCAACACAAAGG (20 bp)
β-actin	ACAGCAGTTGGTTGGAGCAA (20 bp)	ACGCGACCATCCTCCTCTTA (20 bp)

IL-6, interleukin-6; IL-1β, interleukin-1β; TNF-α, tumor necrosis factor; PPARα, peroxisome proliferator-activated receptor alpha; FAS, fatty acid synthase; PPAR*γ*, peroxisome proliferator-activated receptor gamma; SREBP1c, sterol regulatory element-binding protein 1c; CPT-1, carnitine palmitoyl transferase-1; LXR, liver X receptor.

### Gut microbiota analysis

Sequencing and analysis of the cecal microbiota were conducted with reference to previous methods (14). Venn diagram was used to represent the intersection relation of microbial operational taxonomic unit (OTU) between groups using the R package. Canonical correspondence analysis (CCA) was conducted to exhibit the correlation between the environmental factors and microbial community. Pearson correlation analysis was used to analyze the correlation between the flora and the environment.

### Statistical analysis

Data analysis was performed with SPPS (version 22.0) and experimental data were presented as means ± standard error of mean (SEM). The two-tailed Student’s *t*-test and one-way ANOVA followed by a Duncan *post hoc* test were used to compare differences between two groups and between multiple groups, respectively. Results were defined significant as *p* < 0.05. Omicsmart with the online platform^1^ was used to analyze gut microbiota.

## Results

### Determination of the main active ingredients in green tea powder

The main active ingredients of green tea powder included tea polyphenols, tea dietary fiber, and amino acids (Table 2). The polyphenol content of tender tea powder was (20.27 ± 1.02)%, which was significantly higher than that of the medium tender tea and coarse tea (*p* < 0.05). Among different catechins, EGCG content was the highest (13.53 ± 0.78)%. The EC and ECG contents were similar. The polyphenol and catechin contents of tea were significantly different in tea powder with different qualities. The total dietary fiber content of tea powder with different qualities was (24.36 ± 0.54)%, (29.01 ± 0.82)%, and (37.62 ± 1.27)%, which is mainly composed of insoluble dietary fiber. The dietary fiber content of coarse green tea powder was significantly higher than those of tender and medium tender tea powders (*p* < 0.05). The free amino acid and theanine contents of tender tea powder were the highest, which were significantly higher than those of the medium tender tea and coarse tea (*p* < 0.05).

**TABLE 2:** The main active constituents of green tea powder.

Main active constituents of green tea powder	Tender tea powder (T)	Medium tender tea powder (M)	Coarse tea powder (C)
Tea polyphenol (%)	20.27 ± 1.02^a^	18.33 ± 0.89^b^	17.03 ± 1.13^c^
Total dietary fiber (%)	24.36 ± 0.54^c^	29.01 ± 0.82^b^	37.62 ± 1.27^a^
Soluble dietary fiber (%)	1.44 ± 0.06^b^	1.87 ± 0.12^b^	2.85 ± 0.07^a^
Insoluble dietary fiber (%)	22.92 ± 0.54^c^	27.14 ± 0.33^b^	34.77 ± 0.27^a^
Cellulose (%)	9.02 ± 0.14^c^	11.11 ± 0.22^b^	13.83 ± 0.28^a^
Hemicellulose (%)	4.31 ± 0.18^b^	5.33 ± 0.17^b^	7.33 ± 0.20^a^
Lignin (%)	7.32 ± 0.21^c^	9.37 ± 0.26^b^	11.29 ± 0.24^a^
Pectin (%)	0.78 ± 0.03^b^	0.90 ± 0.04^b^	1.66 ± 0.11^a^
Free amino acids (%)	3.49 ± 0.22^a^	2.6 ± 0.56^b^	2.11 ± 0.33^b^
Theanine (%)	2.02 ± 0.17^a^	1.54 ± 0.09^b^	1.09 ± 0.07^c^
**Tea polyphenol**			
EGC (μg/mL)	7.40 ± 0.76^a^	5.60 ± 0.49^b^	4.29 ± 1.10^c^
EC (μg/mL)	3.66 ± 0.89^a^	3.12 ± 0.75^b^	2.56 ± 0.50^c^
EGCG (μg/mL)	13.53 ± 0.78^a^	11.07 ± 0.66^b^	9.02 ± 0.29^c^
ECG (μg/mL)	3.98 ± 0.08^a^	3.01 ± 0.12^b^	2.67 ± 0.14^c^

Different letters (a–c) show significant difference (*p* < 0.05).

### Tea powder prevented obesity and dyslipidemia caused by high-fat diet

**FIGURE 1:**
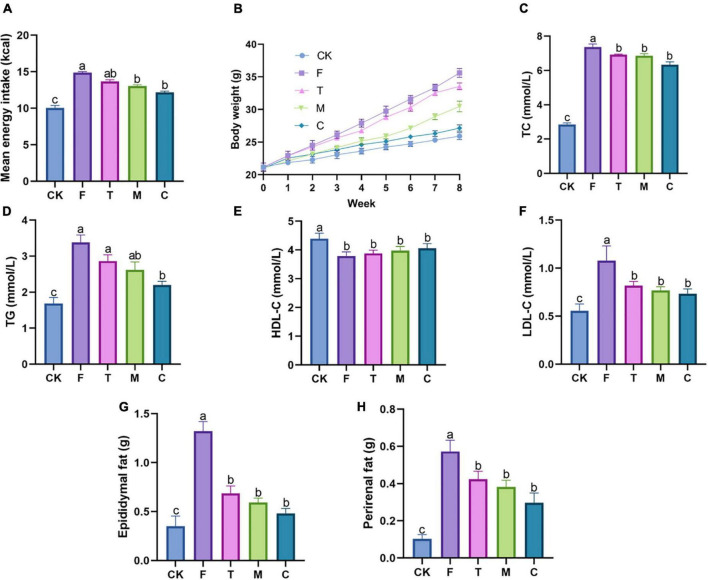
Different qualities of green tea powder reduced body weight and improved dyslipidemia. **(A)** Mean energy intake. **(B)** Body weight variation trend. **(C)** Serum TC levels. **(D)** Serum TG levels. **(E)** Serum HDL-C levels. **(F)** Serum LDL-C levels. **(G)** Epididymal fat weight. **(H)** Perirenal fat weight. Different letters (a–c) showed significant difference (*p* < 0.05).

As shown in Figure 1A, the energy intake of mice was compared between each group during the 8-week intervention. Compared with the F group, the energy intakes of the tea powder groups were reduced, suggesting that tea powder alleviated obesity partially by inhibiting mice appetite. In particular, coarse tea powder was able to reduce their energy intake significantly (*p* < 0.05). The HFD mice groups gained much more weight than the CK group (Figure 1B). Compared with the F group, the body weights of the mice induced by HFD were decreased under tea powder intervention. Notably, the coarse tea powder group significantly decreased the body weights (*p* < 0.05).

As shown in Figures 1C–F, tea powder intervention decreased the levels of serum TC, TG, and LDL-C in the HFD obese mice. The coarse tea powder group had the most significant effect on anti-hyperlipidemia (*p* < 0.05). Serum HDL-C levels were not affected by the green tea powder treatment.

As shown in Figures 1G,H, HFD significantly increased the weight of epididymal fat and perirenal fat, whereas these changes were substantially mitigated by green tea powder intervention. The administration of coarse tea powder significantly decreased the epididymal fat weight (*p* < 0.05), which was comparable to that of the CK group.

### Histological analysis of the liver and colonic tissues

**FIGURE 2:**
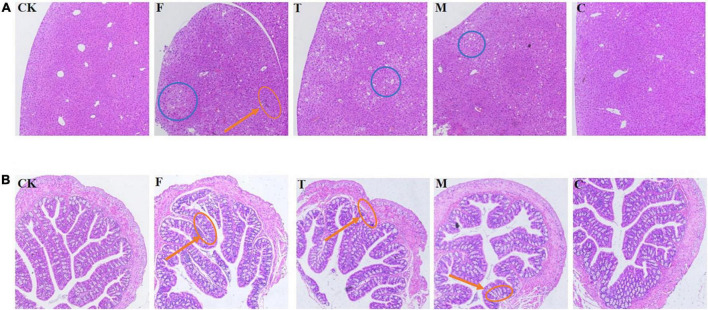
Histological examination of the liver and colon tissues (H&E, 100 ×). **(A)** Histological examination of liver tissue. **(B)** Histological examination of the colon tissue. Red callouts indicated inflammation; blue callouts indicated fat deposits.

As shown in Figure 2, through the observation of liver slices, the liver cells in the high-fat model group had a little inflammatory infiltration, while the green tea powder group significantly reversed this phenomenon. For the colon tissue, the intestinal mucosal folds in the high-fat model group were obviously damaged and shed, and severe atrophy of intestinal villi. The coarse powder group showed better intestinal integrity and the intestinal epithelial cells were closely arranged. Moreover, the colon tissues of the high-fat diet induced obesity mice appeared to marked inflammatory infiltrate. Tea powder effectively reduced inflammatory infiltrate. These results indicated that coarse powder was more effective in maintaining the integrity of the intestine and reducing inflammation.

### Effects of tea powder on the systemic inflammatory response of the high-fat diet-induced obesity mice

**FIGURE 3:**
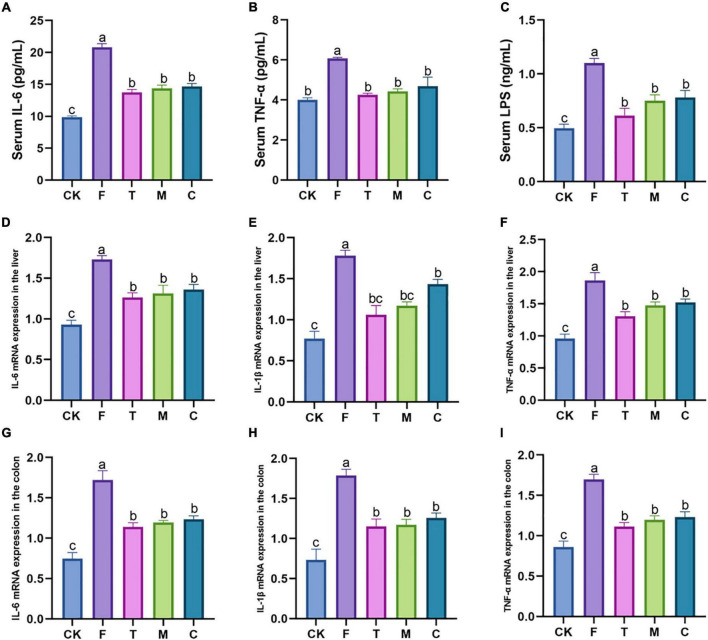
Different qualities of green tea powder reduced the systemic inflammation response of high-fat diet induced obesity in mice. **(A)** Serum IL-6 concentrations. **(B)** Serum TNF-α concentrations. **(C)** Serum LPS concentrations. **(D)** IL-6 mRNA expression levels in the liver. **(E)** IL-1β mRNA expression levels in the liver. **(F)** TNF-α mRNA expression levels in the liver. **(G)** IL-6 mRNA expression levels in the colon. **(H)** IL-1β mRNA expression levels in the colon. **(I)** TNF-α mRNA expression levels in the colon. Different letters (a–c) showed significant difference (*p* < 0.05).

As shown in Figures 3A–C, tea powder significantly alleviated the inflammatory response caused by the HFD, and significantly reduced the serum IL-6, TNF-α, and LPS concentrations (*p* < 0.05). We also further examined the effect of tea powder on the mRNA gene expression levels with the inflammation of the liver and colon in the HFD-induced obesity mice (Figures 3D–I). We found the results trend was consistent with that blood. Tea powder significantly reduced the mRNA expression levels of IL-6, IL-1β, and TNF-α in the liver and colon of the high-fat diet fed mice, and three kinds of tea powder with different qualities did not have significant differences.

### Effects of tea powder on the oxidative stress in the liver and colon of the high-fat diet-induced obesity mice

As shown in Table 3, green tea powder intervention could improve the oxidative stress state of the HFD-induced obesity mice, which elevated the antioxidant enzyme activities in the liver and colon, including CAT, SOD, and GSH levels. Meanwhile, tea powder decreased MDA levels. The tender tea powder (T) group showed the best effect (*p* < 0.05) by improving the oxidative stress state in the liver and colon of the HFD-induced obesity mice.

**TABLE 3:** Effects of green tea powder on the oxidative stress state associated indexes in the liver and colon of the high-fat diet induced obesity mice.

Tissues	Groups	SOD (U/mg)	GSH (μ mol/g)	CAT (U/mg)	MDA (nmol/mg)
Liver	CK	18.47 ± 1.97^a^	1.36 ± 0.22^a^	59.02 ± 3.39^a^	0.51 ± 0.08^c^
	F	13.88 ± 2.03^c^	0.78 ± 0.08^c^	55.74 ± 2.60^b^	0.88 ± 0.12^a^
	T	17.27 ± 1.53^b^	1.24 ± 0.12^b^	58.39 ± 4.46^a^	0.57 ± 0.03^c^
	M	16.93 ± 0.89^b^	1.18 ± 0.16^b^	58.74 ± 5.53^a^	0.61 ± 0.07^b^
	C	15.47 ± 0.65^bc^	1.09 ± 0.20^b^	57.70 ± 4.56^a^	0.65 ± 0.04^b^
Colon	CK	8.53 ± 0.82^a^	0.71 ± 0.10^a^	20.12 ± 4.14^a^	0.42 ± 0.04^c^
	F	5.61 ± 0.90^c^	0.32 ± 0.06^c^	15.23 ± 3.33^b^	0.68 ± 0.09^a^
	T	7.56 ± 0.21^b^	0.57 ± 0.11^b^	19.08 ± 2.28^a^	0.45 ± 0.07^c^
	M	7.02 ± 0.53^b^	0.49 ± 0.04^b^	18.89 ± 4.20^a^	0.55 ± 0.06^b^
	C	6.27 ± 0.36^bc^	0.44 ± 0.20^bc^	18.37 ± 2.91^a^	0.59 ± 0.03^b^

Different letters (a–c) show significant difference (*p* < 0.05).

### Effects of green tea powder on the mRNA gene expression levels associated with the lipid metabolism of the liver in the high-fat diet-induced obesity mice

**FIGURE 4:**
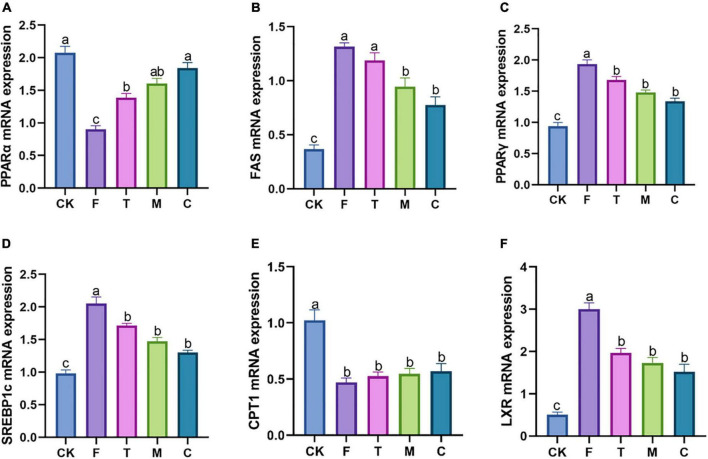
Different qualities of green tea powder improved the lipid metabolism related mRNA genes expression in the high-fat diet induced obesity mice. **(A)** PPARα mRNA expression levels. **(B)** FAS mRNA expression levels. **(C)** PPAR*γ* mRNA expression levels. **(D)** SREBP1c mRNA expression levels. **(E)** CPT-1 mRNA expression levels. **(F)** LXR mRNA expression levels. Different letters (a–c) showed significant difference (*p* < 0.05).

The HFD group (F) increased the relative expression levels of the adipogenic genes compared with the CK group (Figure 4). In contrast to the F group, coarse tea powder could significantly downregulate the mRNA levels of FAS, PPAR-*γ*, LXR, and SREBP1c (*p* < 0.05), which inhibited the expression of adipogenic genes. Coarse tea powder also increased the mRNA levels of PPAR-α.

### Effects of tea powder on gut microbiota

As shown in Table 4, the alpha diversity, including richness (observed species and Chao1) and diversity (Shannon and PD-tree), of the cecal microbiota in mice were calculated. The HFD group has relatively lower richness and diversity compared with the LFD group. Compared with the F group, the intervention of green tea powder with different qualities significantly increased microbial richness and diversity in the cecum. The coarse tea powder group (C) has the highest richness (Observed species, 612 ± 41; Chao 1, 721.9 ± 41.8) and diversity (Shannon, 5.7 ± 0.6; PD-tree, 68.7 ± 7.6) in the treatment group.

**TABLE 4:** Alpha diversity indexes of cecal microbiota associated with mice from CK, F, T, M, and C groups.

Groups	Observed species	Chao1	Shannon	PD-tree
CK	652 ± 47	730.2 ± 42.1	6.2 ± 1.1	75.2 ± 8.7
F	472 ± 33	569.3 ± 37.0	5.0 ± 0.9	52.3 ± 9.1
T	502 ± 37	614.4 ± 25.5	5.2 ± 0.8	61.6 ± 5.3
M	547 ± 29	666.0 ± 29.6	5.4 ± 0.4	66.8 ± 8.2
C	612 ± 41	721.9 ± 41.8	5.7 ± 0.6	68.7 ± 7.6

**FIGURE 5:**
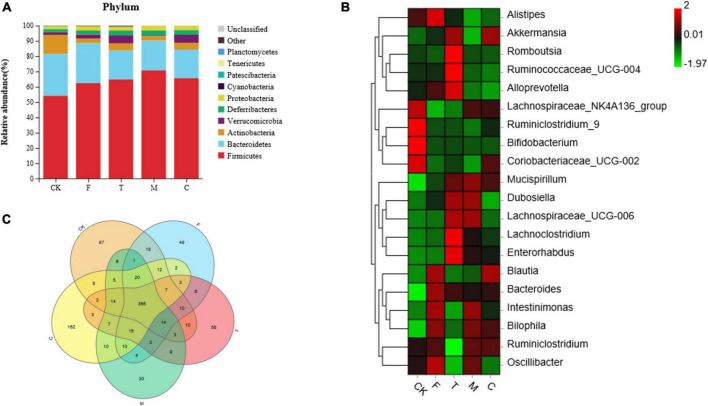
Green tea powder with different qualities modulated the gut microbiota in high-fat diet induced obesity mice. **(A)** Cecal microbiota composition at the phylum level. **(B)** Heatmap of the cecal microbiota at the genus level. **(C)** Venn diagram of intersection relation in the operational taxonomic units (OTUs) of cecal microbiota among groups.

As shown in Figure 5A, the bacterial communities of the ceca of the mice were dominated mainly by *Firmicutes*, *Bacteroidetes*, and *Actinobacteria*. Compared with the CK group, there was an apparent reduction in the abundance of *Actinobacteria*, and an increase in the abundance of *Proteobacteria* in the HFD group (*p* < 0.05). Compared with the F group, the abundance of *Actinobacteria* was significantly enhanced and the abundances of *Bacteroidetes* and *Proteobacteria* were significantly reduced in the green tea powder intervention groups (*p* < 0.05).

As shown in Figure 5B, compared with the F group, green tea powder downregulated the abundances of *Alistipes*, *Bacteroides*, and *Intestinimonas*, and upregulated the abundances of *Akkermansia* and *Mucispirillum*. Especially, the tender tea powder group (T) significantly decreased the abundances of *Blautia*, *Bilophila*, and *Oscillibacter*, and increased the abundances of *Alloprevotella*, *Lachnoclostridium*, *Romboutsia*, and *Ruminococcaceae*_UCG-004 (*p* < 0.05). The coarse tea powder group (C) significantly decreased the abundance of *Dubosiella* and increased the abundances of the *Lachnospiraceae*_NK4A136 group and *Coriobacteriaceae*_UCG-002.

As shown in Figure 5C, the Venn diagram illustrated the intersection relation in the OTUs of cecal microbiota within the groups. A total of 366 (78.2%) OTUs were shared among all the groups. The CK, F, T, M, and C groups showed 87, 49, 56, 30, and 162 unique OTUs out of the total OTUs, respectively.

**FIGURE 6:**
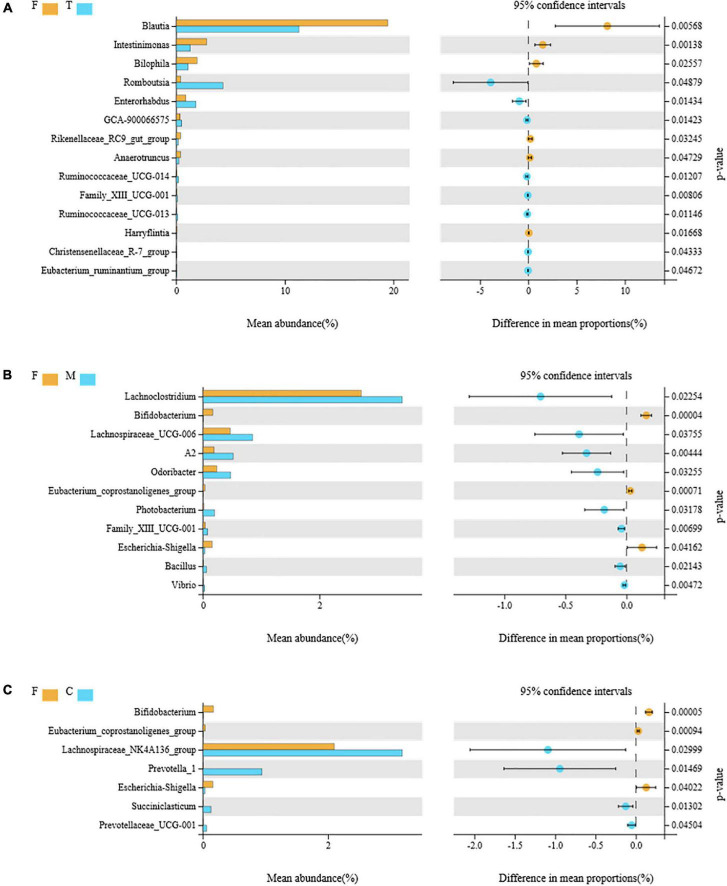
Comparison of the differential intestinal bacteria between the F group and green tea powder group at the genus level (*p* < 0.05). **(A)** Comparison of the differential intestinal bacteria between the F group and T group. **(B)** Comparison of the differential intestinal bacteria between the F group and M group. **(C)** Comparison of the differential intestinal bacteria between the F group and C group.

To compare the differences in microflora between the F group and each of the tea powder groups, a Welch’s *t*-test was used for the analysis. When *p* < 0.05, it was considered significant (Figure 6).

Eight genera (Figure 6A), *Romboutsia*, *Enterorhabdus*, GCA-90006657, *Ruminococcaceae*_UCG-014, Family_XIII_U CG-001, *Ruminococcaceae*_UCG-013, the *Christensenellaceae*_ R-7_group, and the *Eubacterium*_*ruminantium*_group, were overrepresented in the T (tender tea powder) group, while the abundances of six genera, *Blautia*, *Intestinimonas*, *Bilophila*, the *Rikenellaceae*_ RC9_ gut_ group, *Anaerotruncus*, and *Harryflintia* decreased significantly in the T group.

Eight genera (Figure 6B), *Lachnoclostridium*, *Lachnospiraceae*_UCG-006, A2, *Odoribacter*, *Photobacterium*, Family_XIII_UCG-001, *Bacillus*, and *Vibrio*, were overrepresented in the M (medium tender tea powder) group, while the abundances of three genera, *Bifidobacterium*, *Eubacterium*_*coprostanoligenes*_group, and *Escherichia*-*Shigella* decreased significantly in the M group.

Four genera (Figure 6C), *Lachnospiraceae*_NK4A136_ group, *Prevotella*_1, *Succiniclasticum*, and *Prevotellaceae*_UCG-001 were overrepresented in the C (coarse tea powder) group, while the abundances of three genera, *Bifidobacterium*, *Eubacterium*_*coprostanoligenes*, and *Escherichia*-*Shigella* decreased significantly in the C group.

**FIGURE 7:**
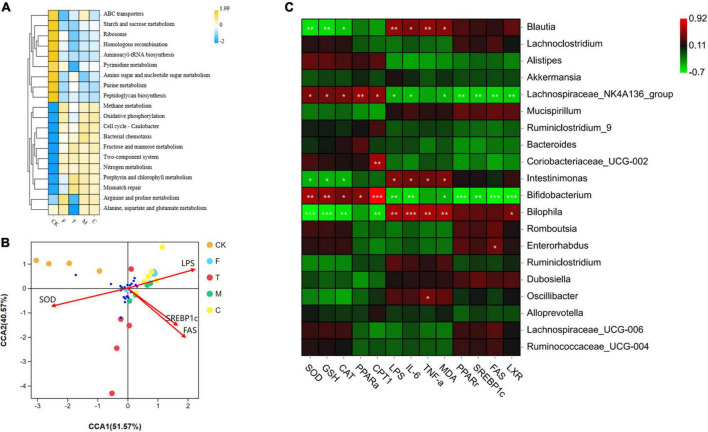
**(A)** Comparison of bacterial functions predicted by Tax4Fun among groups. **(B)** Canonical correspondence analysis (CCA) analysis of between the cecal microbiota and environmental factors. **(C)** Heatmap of Pearson correlation analysis between specific microbiota and obesity related indexes, including SOD, GSH, CAT, PPARα, CPT1, LPS, IL-6, TNF-α, MDA, PPAR*γ*, SREBP1c, FAS, and LXR. The green color represented negative correlation, and red color represented positive correlation. A significant correlation is labeled by * *p* < 0.05, **p < 0.01, ***p < 0.001.

As shown in Figure 7A, the microbial functional analysis indicate that compared with the F group, three tea powder groups showed increased ABC transporters, starch and sucrose metabolism, fructose and mannose metabolism, amino sugar and nucleotide sugar metabolism, porphyrin and chlorophyll metabolism, and mismatch repair.

To investigate the relationship between cecal microorganisms and environmental factors, the CCA model (Figure 7B) was conducted along with four main environmental factors, SOD, LPS, SREBP1c, and FAS. CCA1 and CCA2 accounted for 51.57 and 40.57%, respectively. Pearson correlation analysis (Figure 7C) was conducted to further understand the correlation between environmental factors, including SOD, GSH, CAT, PPAR-α, CPT1, LPS, IL-6, TNF-α, MDA, PPAR-*γ*, SREBP1c, FAS, LXR, and specific microorganisms. The *Lachnospiraceae*_NK4A136_group and *Bifidobacterium* were positively correlated with SOD, GSH, CAT, PPAR-α, and CPT1. *Intestinimonas* and *Bilophila* were negatively correlated with SOD, GSH, and CAT. *Blautia* and *Bilophila* were positively correlated with LPS, IL-6, TNF-α, and MDA. *Coriobacteriaceae*_UCG-002 was positively correlated with CPT1. *Enterorhabdus* was positively correlated with FAS. *Oscillibacter* was positively correlated with TNF-α.

## Discussion

We systematically explored the effects of different qualities of green tea powder on HFD-induced obesity mice. These results suggest that different qualities of green tea powder had different concentrations of active components, while multiple active constituents of the tea powders might serve different functions in obesity. Cecal microbiota profiling of multiple bacteria taxa exhibited diverse modulatory effects for different qualities of green tea powder.

The polyphenol content of tender tea powder was significantly higher than those of the medium tender tea and coarse tea. The synthesis of tea polyphenols requires the participation of a variety of enzymes, such as phenylalanine ammonium lyase (PAL), chalcone synthase (CHS), and dihydroflavonol reductase (DFR). The PAL and CHS genes are highly expressed in the tender leaves of tea; however, these two genes can hardly be detected in the coarse and old leaves of tea (15). The tender degree of tea significantly affected the dietary fiber content of the tea, and the dietary fiber content of the tea gradually increased with the decrease in tenderness (16), which is consistent with the results obtained in this study. The theanine content of tender tea powder was the highest, which has the characteristics of fresh sweet and high fresh fragrance (17, 18). The cultivation conditions and climate may affect the content of theanine; spring tea with tender bud leaves has a relatively high content of theanine (19), which is consistent with our results.

Different qualities of green tea powder supplementation could remarkably suppress body weight gain, and reduce serum TC, TG, and LDL-C of HFD-induced obesity mice. Coarse tea powder suppressed body weight and improved hyperlipidemia. In addition, coarse tea powder significantly reduced the mRNA expression of lipid synthesis genes, such as SREBP1c and FAS. Analysis of the reasons may be that coarse tea powder has more dietary fiber and polysaccharides. A study indicated that the tea polysaccharides of coarse tea powder were twice as high as that of the tender tea powder, and treating diabetes with coarse tea powder might be related to its high content of tea polysaccharides (4). As a gene associated with energy metabolism, FAS is responsible for fatty acid synthesis and TG accumulation. LXR is a nuclear receptor that plays a vital role in the lipid synthesis of the liver (20). SREBP-1c can regulate the expression of lipid synthesis enzymes (21). PPAR-α has been reported to decreases TG and LDL-C levels through promoting β-oxidation and lipolysis (22). In our results, the mRNA expression of FAS, PPAR-*γ*, and SREBP1c was significantly reduced and the mRNA expression of PPAR-α was significantly increased by coarse tea powder. Therefore, the results show that the anti-obesity benefit of coarse tea powder might be partly attributed to the improvement of lipid metabolism.

Green tea powder could inhibit high-fat diet induced significant increment in pro-inflammatory cytokines, including TNF-α, IL-6, and LPS, in the serum, as evidenced by decreased mRNA levels of IL-6, IL-1β, and TNF-α in the liver and colon. Altogether, green tea powder intervention could improve high-fat diet-driven inflammation.

Obesity is accompanied with oxidative stress damage. Therefore, the antioxidant capacity of green tea powder was also assessed. Tender tea powder contains more tea polyphenols, including EGCG, EGC, ECG, and EC, demonstrating very good antioxidant activity on account of their ability to scavenge free radicals and increase the activity of antioxidant enzymes. The level of SOD, GSH, and CAT was significantly increased and the level of lipid peroxidation product MDA was significantly decreased under tender tea powder intervention, which indicated that tender tea powder might improve oxidative stress states in obese mice.

The gut microbiota has been regarded to be a potential intervention target for the alleviation of obesity. From the cecal microflora of mice, the richness and diversity were decreased by the HFD, while green tea powder ameliorated the reduction. Some studies have shown that the lower richness and diversity of the intestinal microbiota is associated with obesity. In addition, compared with coarse tea powder, tender tea powder contains a relatively higher content of tea polyphenols. Tea polyphenols are well known for their excellent antibacterial effects and have certain inhibitions on microorganisms, including gram-negative and -positive bacteria (23). Therefore, it is reasonable for the tender tea powder group to have low bacterial abundance and diversity.

Green tea powder could proliferate specific beneficial microbiota and decrease some pathogenic bacteria in the cecum of HFD-induced obesity mice. Compared with the F group, green tea powder downregulated the abundances of *Alistipes*, *Bacteroides*, and *Intestinimonas*, and upregulated the abundances of *Akkermansia* and *Mucispirillum*. *Alistipes* are reported to be positively associated with body weight, TC, and TG (24). *Bacteroides*, such as *Bacteroides fragilis* and *Bacteroides vulgatus*, which are potentially harmful bacteria, might cause infection and inflammation (25). In the inflammatory animal model, a higher abundance of *Intestinimonas* were indicated (26). *Akkermansia* has been reported to be reversely associated with inflammation, obesity, and diabetes (27). *Mucispirillum* could protect mice against pathogenic *Salmonella* colitis (28).

The coarse tea powder group (C) significantly decreased the abundance of *Dubosiella*, and increased the abundances of the *Lachnospiraceae*_NK4A136 group and *Coriobacteriaceae*_UCG-002. *Lachnospiraceae* is known to have an anti-inflammatory effect and can generate short-chain fatty acids (SCFAs). SCFAs can exert multifaceted functions, decrease the pH of the large intestine, and inhibit the pathogenic *Coriobacteriaceae*, the potential target for preventing metabolic syndrome (29). *Dubosiella* has been suggested to be associated with a diet high in dietary advanced glycation end products, and eventually caused inflammation (30).

The tender tea powder group (T) significantly decreased the abundances of *Blautia*, *Bilophila* and *Oscillibacter*, and increased the abundances of *Alloprevotella*, *Lachnoclostridium*, *Romboutsia*, and *Ruminococcaceae*_UCG-004. In a population study trial, *Blautia* has been indicated that it was associated with obesity and fat deposits (31). *Bilophila* significantly exacerbated HFD-induced inflammatory response and metabolic related disease (32). *Oscillibacter* can decreased the levels of the proteins related to gut mucosal barrier, which are considered to have strongly associated with HFD-induced obesity (33). *Alloprevotella* are SCFA producers, such as acetic and succinic acids, which can maintain the gut epithelial barrier and modulate immunity function (34). *Romboutsia* can metabolize and utilize carbohydrates and amino acids (35). *Ruminococcaceae* could produce SCFAs and alleviate inflammation and obesity (36). The function of *Lachnoclostridium* in preventing obesity has rarely been reported, more research will be needed to clarify this phenomenon.

Intestinal metabolites also could affect the obesity caused by a high-fat diet. In our study, medium tender tea powder and coarse tea powder reduced the mean energy intake of the HFD-induced obesity mice. It was speculated that the medium tender tea and coarse tea powder improved obesity and lipid metabolism through SCFAs. Evidence has suggested that SCFAs could affect the appetite and hormone secretion by binding to the G protein-coupled receptors in intestinal tissues, which could regulate the energy metabolism of the host (37). For example, a gut satiety hormone called peptide YY could play a role through brain appetite suppression, increased intestinal transit and decreased energy intake. In addition, high dietary fiber food active substances have showed to the ability to decrease the energy intake and subjective appetite (38). Therefore, medium tender tea powder and coarse tea powder alleviated the obesity partly owed to the reduced the food intake and increased the satiety.

Our results revealed that some specific bacteria are correlated with environmental factors. Therefore, the results show a significant association between these bacteria and related parameters of obesity, inflammation, oxidative stress state, and mRNA expression of lipid metabolism, indicating these bacteria might be strongly associated with prevention of the obesity development. More studies will be needed to investigate whether these specific bacteria will become new biomarkers for obesity in the future.

In summary, our present study shows that all three types of tea powder with different qualities could reduce body weight and decrease serum TC, TG, and LDL-C. However, green tea powder of different qualities exhibited diverse modulatory effects and mechanisms against obesity. The anti-obesity diverse modulation effects and mechanism of green tea powder involved the differentiation of gut microbiota, regulation of oxidative stress state, regulation of lipid metabolism and affection of food intake. Our findings provide a new perspective and valuable information for the utilization and selection of green tea powder.

## Data availability statement

The original contributions presented in this study are included in the article/supplementary material, further inquiries can be directed to the corresponding author.

## Ethics statement

The animal study was reviewed and approved by the Animal Experimental Center of Nankai University.

## Author contributions

JW and LD performed the experiments, analyzed the data, and prepared this study. J-QH and AL analyzed the data and modified the format in this study. Y-Y-FW and B-WZ were responsible for methodology. BP and J-ML revised the manuscript. SW conceived and designed the experiment. All authors have read and agreed to the published version of the manuscript.

## Footnotes

1
http://www.omicsmart.com

## Abbreviations

HFD: high-fat diet
TC: total cholesterol
TG: triglyceride
LDL-C: low-density lipoprotein cholesterol
HDL-C: high-density lipoprotein cholesterol
EGCG: epigallocatechin gallate
EC: epicatechin
ECG: epicatechin gallate
EGC: epigallocatechin
HPLC: high-performance liquid chromatography
H&E: hematoxylin and eosin
LPS: lipopolysaccharides
ELISA: enzyme-linked immunosorbent assay
TNF-α: tumor necrosis factor-alpha
IL-6: interleukin-6
IL-1β: interleukin-1β
PPARα: peroxisome proliferator-activated receptor alpha
FAS: fatty acid synthase
PPAR*γ*: peroxisome proliferator-activated receptor gamma
SREBP1c: sterol regulatory element-binding protein 1c
CPT-1: carnitine palmitoyl transferase-1
LXR: liver X receptor
ELISA: enzyme-linked immunosorbent assay
GSH: glutathione
MDA: malondialdehyde
SOD: superoxide dismutase
CAT: catalase
CCA: canonical correspondence analysis
SEM: standard error of mean
PAL: phenylalanine ammonium lyase
CHS: chalcone synthase
DFR: dihydroflavonol reductase
SCFAs: short chain fatty acids.
